# Hyperglycemia induces key genetic and phenotypic changes in human liver epithelial HepG2 cells which parallel the Lepr^db^/J mouse model of non-alcoholic fatty liver disease (NAFLD)

**DOI:** 10.1371/journal.pone.0225604

**Published:** 2019-12-05

**Authors:** Robin C. Su, Apurva Lad, Joshua D. Breidenbach, Thomas M. Blomquist, William T. Gunning, Prabhatchandra Dube, Andrew L. Kleinhenz, Deepak Malhotra, Steven T. Haller, David J. Kennedy

**Affiliations:** 1 Department of Medicine, The University of Toledo College of Medicine and Life Sciences, Toledo, Ohio, United States of America; 2 Department of Pathology, The University of Toledo College of Medicine and Life Sciences, Toledo, Ohio, United States of America; 3 Department of Medical Microbiology and Immunology, The University of Toledo College of Medicine and Life Sciences, Toledo, Ohio, United States of America; Universita degli Studi di Catania, ITALY

## Abstract

Non-alcoholic fatty liver disease (NAFLD) is a growing global health concern. With a propensity to progress towards non-alcoholic steatohepatitis (NASH), cirrhosis, and hepatocellular carcinoma, NAFLD is an important link amongst a multitude of comorbidities including obesity, diabetes, and cardiovascular and kidney disease. As several *in vivo* models of hyperglycemia and NAFLD are employed to investigate the pathophysiology of this disease process, we aimed to characterize an *in vitro* model of hyperglycemia that was amenable to address molecular mechanisms and therapeutic targets at the cellular level. Utilizing hyperglycemic cell culturing conditions, we induced steatosis within a human hepatocyte cell line (HepG2 cells), as confirmed by electron microscopy. The deposition and accumulation of lipids within hyperglycemic HepG2 cells is significantly greater than in normoglycemic cells, as visualized and quantified by Nile red staining. Alanine aminotransferase (ALT) and alkaline phosphatase (ALP), diagnostic biomarkers for liver damage and disease, were found to be upregulated in hyperglycemic HepG2 cells as compared with normoglycemic cells. Suppression of CEACAM1, GLUT2, and PON1, and elevation of CD36, PCK1, and G6PK were also found to be characteristic in hyperglycemic HepG2 cells compared with normoglycemic cells, suggesting insulin resistance and NAFLD. These *in vitro* findings mirror the characteristic genetic and phenotypic profile seen in Lepr^db^/J mice, a well-established *in vivo* model of NAFLD. In conclusion, we characterize an *in vitro* model displaying several key genetic and phenotypic characteristics in common with NAFLD that may assist future studies in addressing the molecular mechanisms and therapeutic targets to combat this disease.

## Introduction

Liver disease has become a serious health burden globally and is becoming a leading health concern within the United States. In 2004, liver disease became the third leading diagnosis in outpatient care clinics and the second leading cause of mortality among all forms of digestive diseases [1]. Non-alcoholic fatty liver disease (NAFLD) has become the most common form of liver disease in industrialized countries [2]. With a 25% global prevalence, 51% rate of comorbidity with obesity, 22% rate of comorbidity with type 2 diabetes, and a 69% rate of comorbidity with hyperlipidemia, NAFLD is widely pervasive in our global community and is profoundly destructive in its consequences [3].

Although there have been robust research efforts dedicated to studying NAFLD’s pathogenesis, molecular mechanisms, and disease prevention and therapy, knowledge in these areas is still very limited. With several *in vivo* models of NAFLD being established and widely utilized, an *in vitro* model would be particularly advantageous in order to allow for focused, high throughput investigation into molecular mechanisms and therapeutic discovery. Utilizing a diabetes-like induction method, which simulates one of several physiologically relevant pathways of NAFLD pathogenesis, we have characterized a novel, simple, and efficient *in vitro* model that shares many characteristics of NAFLD. We believe that this *in vitro* model will facilitate a wide range of mechanistic and therapeutic discoveries at the cellular level in order to rapidly drive progress in combatting NAFLD globally.

## Materials and methods

### Cell culture

HepG2 human hepatoma cells were obtained from American Type Culture Collection (Manassas, VA, USA, Catalog No. ATCC HB-8065). To simulate normoglycemic conditions, cells were cultured in Eagle’s Minimum Essential Medium (EMEM) (BD Biosciences, San Jose, CA, USA, VWR Catalog No. 76000–922) supplemented with 10% fetal bovine serum (Rocky Mountain Biologicals, Missoula, Montana, USA, Catalog No. FBS-BBT) and 1% penicillin-streptomycin solution (Caisson Labs, Smithfield, UT, USA, Catalog No. PSL01-100ML). To simulate hyperglycemic conditions, cells were cultured in Dulbecco’s Modified Eagle’s Medium (DMEM) (ThermoFisher Scientific, Waltham, MA, USA, Catalog No. 11995065) supplemented with 10% fetal bovine serum (Rocky Mountain Biologicals) and 1% penicillin-streptomycin solution (Caisson Labs). A complete listing of ingredients of the normoglycemic and hyperglycemic media can be found in S1 Table. In order to establish an osmotic control to account for differences in osmolarity between normoglycemic and hyperglycemic media, HepG2 cells were cultured in a third, separate condition with normoglycemic EMEM media (BD Biosciences) with osmolarity adjusted to that of hyperglycemic DMEM media (ThermoFisher Scientific) by the addition of sodium chloride (J.T. Baker, Phillipsburg, NJ, USA, Catalog No. 3624–19), and further supplemented with 10% fetal bovine serum (Rocky Mountain Biologicals) and 1% penicillin-streptomycin solution (Caisson Labs). All cells were grown in T75 flasks at 37°C and 5% CO_2_.

### Histology

HepG2 cells grown in T75 flasks were trypsinized, neutralized in media, and centrifuged in 15ml conicals to obtain cell pellets. Pellets were washed in phosphate buffered saline (PBS) and re-pelleted. PBS was aspirated from the conicals and cells were resuspended in 100ul of 10% neutral buffered formalin. 500ul of liquified histogel was spread within plastic specimen base molds. The 100ul of resuspended cells were quickly and evenly spread throughout the histogel molds. Mixtures were allowed to set for 5 minutes. Specimen molds were transferred to nylon bags, which were placed in specimen cassettes and submerged in 10% neutral buffered formalin for 24 hours. Sample sections were stained with Hematoxylin & Eosin (H&E). Images of H&E stained samples were imaged at 40X using a Olympus CKX53 microscope and Olympus CellSens software (Standard 1.15) (Center Valley, PA, USA).

### Intracellular lipid content quantification and imaging

HepG2 cells were transferred from T75 flasks to 6 well plates. Once 80% confluent, cells were serum starved for 24 hours using phenol red free EMEM (Quality Biological, Gaithersburg, MD, USA, Catalog No. 10128–658) or phenol red free DMEM (Caisson Labs, Smithfield, UT, USA, Catalog No. DML12-500ML) supplemented with 1% penicillin-streptomycin solution. Following serum starvation, cells were washed with PBS. The cells were then stained with Nile red at 1ul per ml of their respective phenol red free EMEM or DMEM media. Stained cells were incubated for 15 minutes. Cells were washed with PBS and placed in their respective phenol red free EMEM or DMEM media. Fluorescence from the Nile red staining was quantitatively measured using the Cytation 5 Cell Imaging Multi-Mode Reader (BioTek, Winooski, VT, USA). Fluorescence was normalized to total protein concentration in order to correct for cell number. Protein concentration determination was completed by the Lowry protein assay.

Cells stained with Nile red were imaged following Cytation 5 quantitative fluorescence measurement and before protein determination. Fluorescent and brightfield images (4X) were captured using the Cytation 5 reader.

### Electron microscopy

For transmission electron microscopy, cell cultures were fixed with 3% glutaraldehyde buffered by 0.2M sodium cacodylate (pH = 7.2) for 30 min. at 37°C and subsequently post-fixed for 2 hrs. at room temperature with 1% osmium tetroxide followed by 1 hr. with saturated aqueous uranyl acetate. Dehydration was carried out through a graded series of ethanols and scrapped from the culture plates with a rubber policeman prior to an exchange of 100% acetone for miscibility with the embedding media. Cells were embedded in Spurr’s resin (Electron Microscope Sciences, Fort Washington, PA) and ultrathin sections were collected on copper 300-mesh support grids. Sections were stained with uranyl acetate and Reynold’s lead citrate, and examined using a FEI Tecnai T20 TEM at 80kV.

### Gene expression analysis

HepG2 cells were transferred from T75 flasks to 6 well plates. Once 80% confluent, cells were serum starved for 24 hours using phenol red free EMEM or DMEM (Quality Biological, Inc., and Caisson Labs, respectively) supplemented with 1% penicillin-streptomycin solution. Following serum starvation, cells were washed with PBS. RNA was isolated utilizing the Qiagen RNeasy Plus Mini Kit (Qiagen, Germantown, MD, USA, Catalog No. 74134) and the Qiagen QIAcube extraction methodology. Approximately 500ng of extracted RNA was used to synthesize cDNA (QIAGEN’s RT2 First Strand Kit, Catalog No. 330401). RT-qPCR was performed utilizing QIAGEN’s Rotor-Gene Q thermo-cycler. Calculation of gene expression was conducted by comparing the relative change in cycle threshold value (ΔCt). Fold change in expression was calculated using the 2-ΔΔCt equation as previously described [4]. The following Taqman primers were used and obtained from Thermo Fisher Scientific: ALT (Hs00193397_m1), ALP (Hs00758162_m1), CD36 (Hs00354519_m1), PCK1 (Hs00159918_m1), G6PC (Hs00609178_m1), SLC2A2 or GLUT2 (Hs01096908_m1), and PON1 (Hs001665557_m1). The tata-box binding protein taqman primer (Hs00427620_m1), also from Thermo Fisher Scientific, was used as the housekeeping gene for normalization of ALT, ALP, CD36, and PON1 taqman gene of interest primers. 18s rRNA from Thermo Fisher Scientific (Catalog No. 4319413E) was used as a housekeeping gene for normalization of transcript expression for PCK1, G6PC, and SLC2A2. CEACAM1 sybr green primers were obtained from Invitrogen with a forward sequence of 5'-TCTACCCTGAACTTTGAAGCCCA-3' and a reverse sequence of 5'-TGAGAGACTTGAAATACATCAGCACTG-3' (Invitrogen, Carlsbad, CA, USA). The GAPDH sybr green primer was used as the housekeeping gene for normalization of CEACAM1. The GAPDH forward sequence used was 5'-ATCCATGACAACTTTGTTATCGTG-3' and reverse sequence used was 5'-ATGACCTGGCCCACAGCCTT-3'. A separate, comprehensive Qiagen Human Hepatotoxicity RT^2^ Profiler Array (Cat. PAHS-093Z) was performed to assess genetic markers of hepatotoxicity, nongenotoxic hepatocarcinogenicity, necrosis, steatosis, and cholestasis.

### MTT and LDH assays

For the determination of cytotoxicity and metabolic activity, we performed the lactate dehydrogenase (LDH) assay and MTT assay, respectively. The LDH assay was carried out according to published protocols with reagents prepared per the instructions therein [5]. Briefly, HepG2 cells were seeded at a density of 8000 cells per well with 100μl of either normoglycemic or hyperglycemic media onto a 96-well plate and cultured at 37°C in a humidified CO_2_ incubator. After 24 hours, Triton X100 was added to half of the wells (for the determination of total LDH) and the plate was mixed on an orbital shaker for 3 minutes. Next, the well plate was centrifuged at 200 X G for 5 minutes and 50μl of supernatant was transferred to new 96 well plates. Assay reagents were mixed with the supernatant and the plate was incubated for 30 minutes in the dark at room temperature. The reaction was stopped with the addition of 1 M acetic acid and the plate was read at 490nm on a spectrophotometer. Cytotoxicity values were calculated using the following equation: % Cytotoxicity = (OD490/(OD490 Triton))X 100.

The MTT assay protocol was adapted from published methods [6]. Briefly, HepG2 cells were seeded at a density of 8000 cells per well with 100μl of normoglycemic or hyperglycemic media onto a 96-well plate and cultured at 37 degrees C in a humidified CO2 incubator. After 24 hours, 10 uL of Thiazolyl Blue Tetrazolium Bromide at 5 mg/mL in PBS was added to each well. The plate was incubated at 37°C for 3 hours, and then half of the media was gently removed and 100μl of 4 mM HCl, 0.1% Nondet P-40 (NP40) isopropanol was added to solubilize the formazan crystals. After thorough mixing, the plate was read on a spectrophotometer at 590nm with a 620nm reference wavelength. Values were calculated using the following equation: Metabolic Activity = ((OD590 Cells-OD590 Media)/(OD620 Cells-OD620 Media)). Next, each metabolic activity value was divided by the average value for normoglycemic grown cells and multiplied by 100 to achieve a percent metabolic activity.

### Animal study

#### Mouse experimental plan

Two groups of mice were utilized in order to compare and contrast our in vitro model with a well established in vivo model of NAFLD. Wild type (WT) C57BL/6J male mice (JAX Stock No. 000664, Black 6, n = 5) were used as a healthy control and B6.BKS(D)-Lepr^db^/J (JAX Stock No. 000697, B6 db) male mice (hereafter referred to as Lepr^db^/J mice, n = 6) on the C57Bl/6J background were used as a NAFLD comparison. All mice were obtained from Jackson Laboratory and were specific pathogen free. Mice were housed in plastic cages, fed a normal chow diet and water *ad libitum*, and were maintained in a well-ventilated room maintained at 23±1°C. Mice were obtained at 10 weeks of age and, at 14 weeks of age, all mice were euthanized by CO_2._ Livers were harvested, with sections frozen directly at -80°C or embedded in OCT compound before being frozen at -80°C. All protocols were approved by the University of Toledo Institutional Animal Care and Use Committee.

#### Histology

Liver sections that were embedded in OCT compound and frozen at -80°C were processed for Oil Red O staining. Lipid vesicles were clearly visualized by brightfield microscopy using a Olympus CKX53 microscope and Olympus CellSens software (Standard 1.15) (Center Valley, PA, USA).

#### Gene expression

RNA extraction, cDNA preparation, and RT-qPCR were all performed utilizing the QIAGEN (Germantown, MD, USA) automated liquid handling workflow system (QIAcube HT and QIAgility). RNA from frozen liver tissue was isolated utilizing the QIAzol/Chloroform extraction methodology. Approximately 500ng of extracted RNA was used to synthesize cDNA (QIAGEN’s RT2 First Strand Kit). RT-qPCR was performed utilizing QIAGEN’s Rotor-Gene Q thermo-cycler. Calculation of gene expression was conducted by comparing the relative change in cycle threshold value (ΔCt). Fold change in expression was calculated using the 2-ΔΔCt equation as previously described [4]. The following Taqman primers were used and obtained from Thermo Fisher Scientific: ALT (Mm00805379_g1), ALP (Mm00475834_m1), and CD36 (Mm00432403_m1). 18s rRNA (Catalog No. 4319413E) from Thermo Fisher Scientific was used as a housekeeping gene for ALT and ALP, while beta-2 microglobulin (Mm00437762_m1) from Thermo Fisher Scientific was used as a housekeeping gene for CD36. CEACAM1 sybr green primers were obtained from Invitrogen with a forward sequence of 5'-AATCTGCCCCTGGCGCTTGGAGCC-3' and a reverse sequence of 5'-AAATCGCACAGTCGCCTGAGTACG-3'. The GAPDH sybr green primer was used as the housekeeping gene for normalization of CEACAM1. The GAPDH forward sequence used was 5'-CCAGGTTGTCTCCTGCGACT-3' and reverse sequence used was 5'-ATACCAGGAAATGAGCTTGACAAAGT-3'. A separate, comprehensive Qiagen Mouse Hepatotoxicity RT^2^ Profiler Array (Cat. PAMM-093Z) was run.

### Statistical analysis

Data are presented as the mean ± s.e.m. Data were analyzed by 2-tailed student’s t-test. A p-value of less the 0.05 was considered to be statistically significant. Statistical analysis was performed using GraphPad Prism software (San Diego, CA).

## Results

### Lipid content is elevated in hyperglycemic HepG2 cells

**Fig 1:**
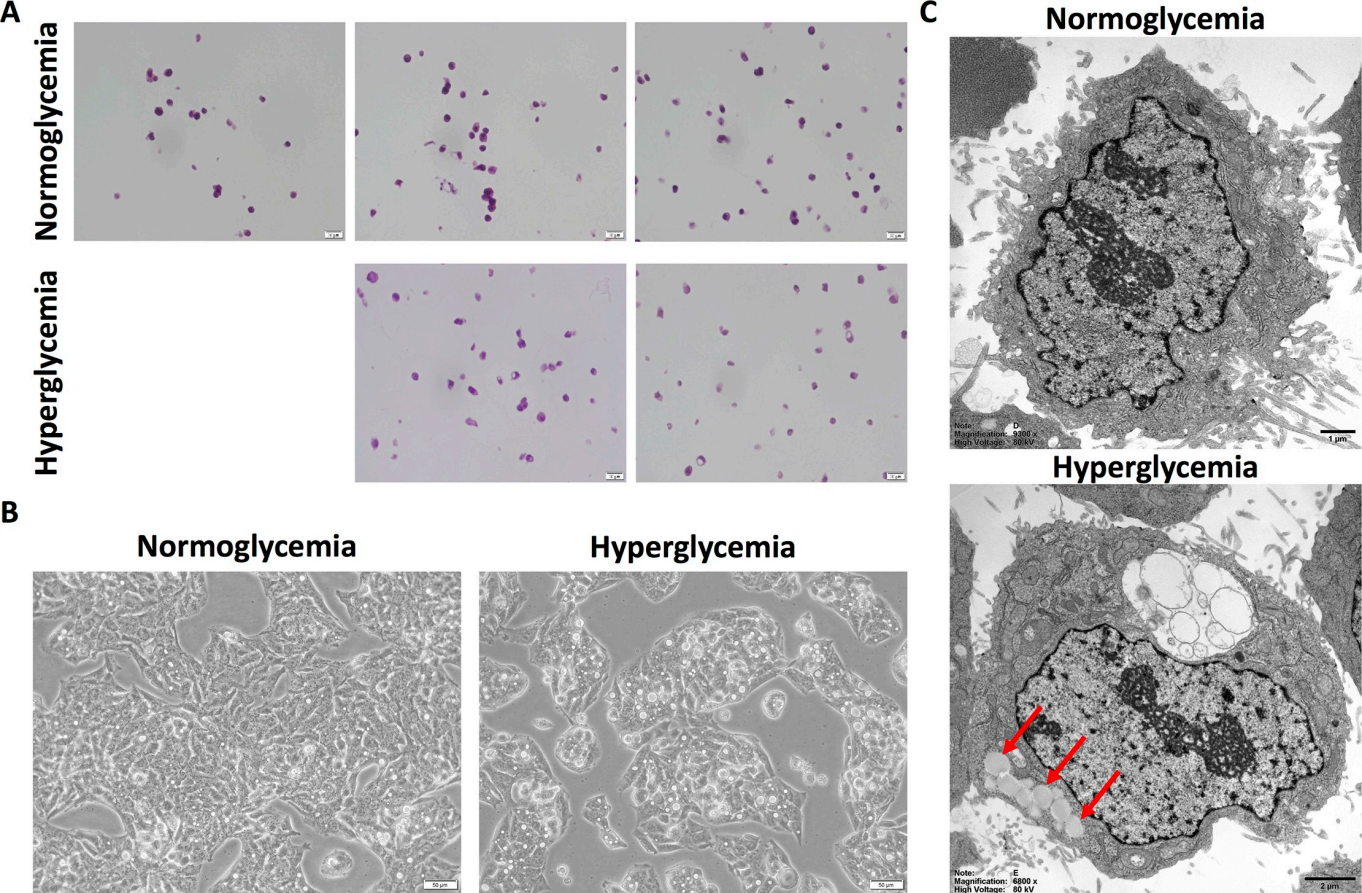
Hyperglycemic HepG2 cells exhibit lipid accumulation. **A.** H&E histology reveals successive passaging of HepG2 cells in hyperglycemic conditions show progressive phenotypic changes as compared with normoglycemic HepG2 cells. **B.** Brightfield microscopy also reveal these phenotypic changes within hyperglycemic HepG2 cells as compared with normoglycemic cells. **C.** Representative cultured cells examined at the ultrastructural level by electron microscopy demonstrate accumulations of numerous lipid vacuoles (red arrows) and water influx (clear cytoplasmic inclusion) in hyperglycemic HepG2 cells in contrast to cells grown in normoglycemic culture media.

Normoglycemic HepG2 cells were split at passage 3 and grown in parallel with continued normoglycemic conditions versus hyperglycemic conditions. By passage 4, hyperglycemic HepG2 cells already began showing phenotypic changes. These findings became even more prominent and pervasive by passage 5. **Fig 1A** shows these progressive changes over several passages with H&E staining. These changes can also be clearly observed with brightfield imaging (**Fig 1B**). As an osmotic control, HepG2 cells were cultured in normoglycemic media with osmolarity adjusted to that of hyperglycemic media by the addition of NaCl. After several passages, no changes in phenotype were observed in these cells as compared with normoglycemic HepG2 cells, but instead retained a similar phenotype to that of normoglycemic HepG2 cells (**S1 Fig**). To further investigate these phenotypic changes, normoglycemic and hyperglycemic HepG2 cells were analyzed using transmission electron microscopy. This ultrastructural imaging revealed profound accumulation and infiltration of microvesicular lipid droplets into hyperglycemic HepG2 cells as compared with normoglycemic HepG2 cells (**Fig 1C**) (red arrows indicate lipid droplets).

**Fig 2:**
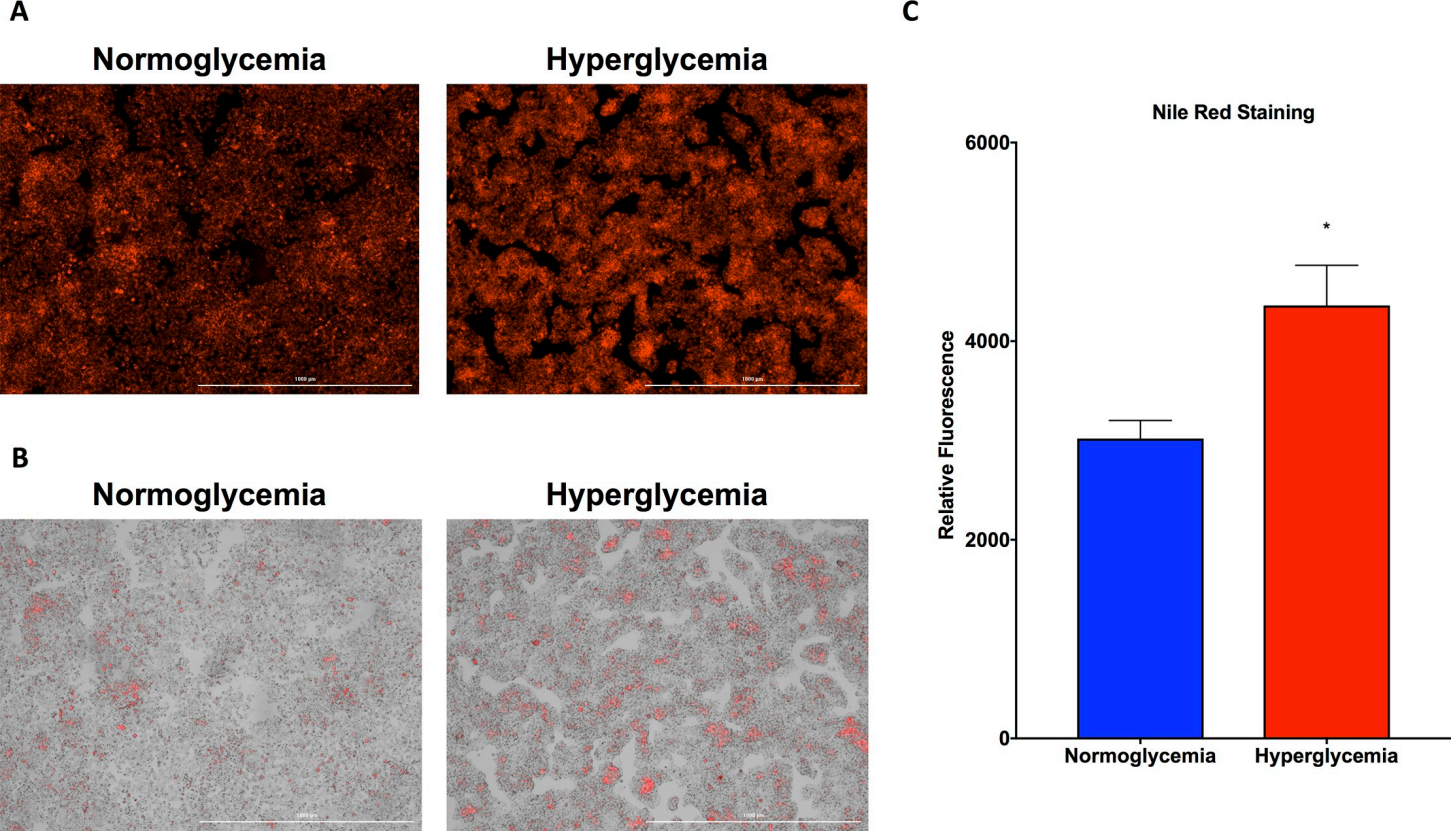
Nile red staining for triglycerides is elevated in hyperglycemic HepG2 cells. **A.** Fluorescent imaging reveals increased Nile red staining in hyperglycemic HepG2 cells as compared with normoglycemic cells. **B.** Brightfield microscopy with fluorescent overlay reveals the accumulation of lipid within hyperglycemic HepG2 cells as compared with normoglycemic cells. **C.** Quantification of Nile red fluorescence reveals elevated levels in hyperglycemic HepG2 cells as compared with normoglycemic cells. Data presented indicate the mean ± SEM (n = 3 wells per group). *p<0.05 vs. normoglycemic group.

Upon Nile red staining for triglycerides, fluorescence intensity was clearly more prominent in the hyperglycemic HepG2 cells, as seen with fluorescent imaging (**Fig 2A**) and brightfield with fluorescent overlay (**Fig 2B**) than in the normoglycemic HepG2 cells. This enhancement in hyperglycemic HepG2 cell Nile red signal was confirmed quantitatively by Cytation 5 fluorescence quantification, which was normalized to protein concentration to correct for differences in cell number (**Fig 2C**).

### Hyperglycemic HepG2 cell gene expression of key biomarkers reflect regulation consistent with that of NAFLD and insulin resistance

**Fig 3:**
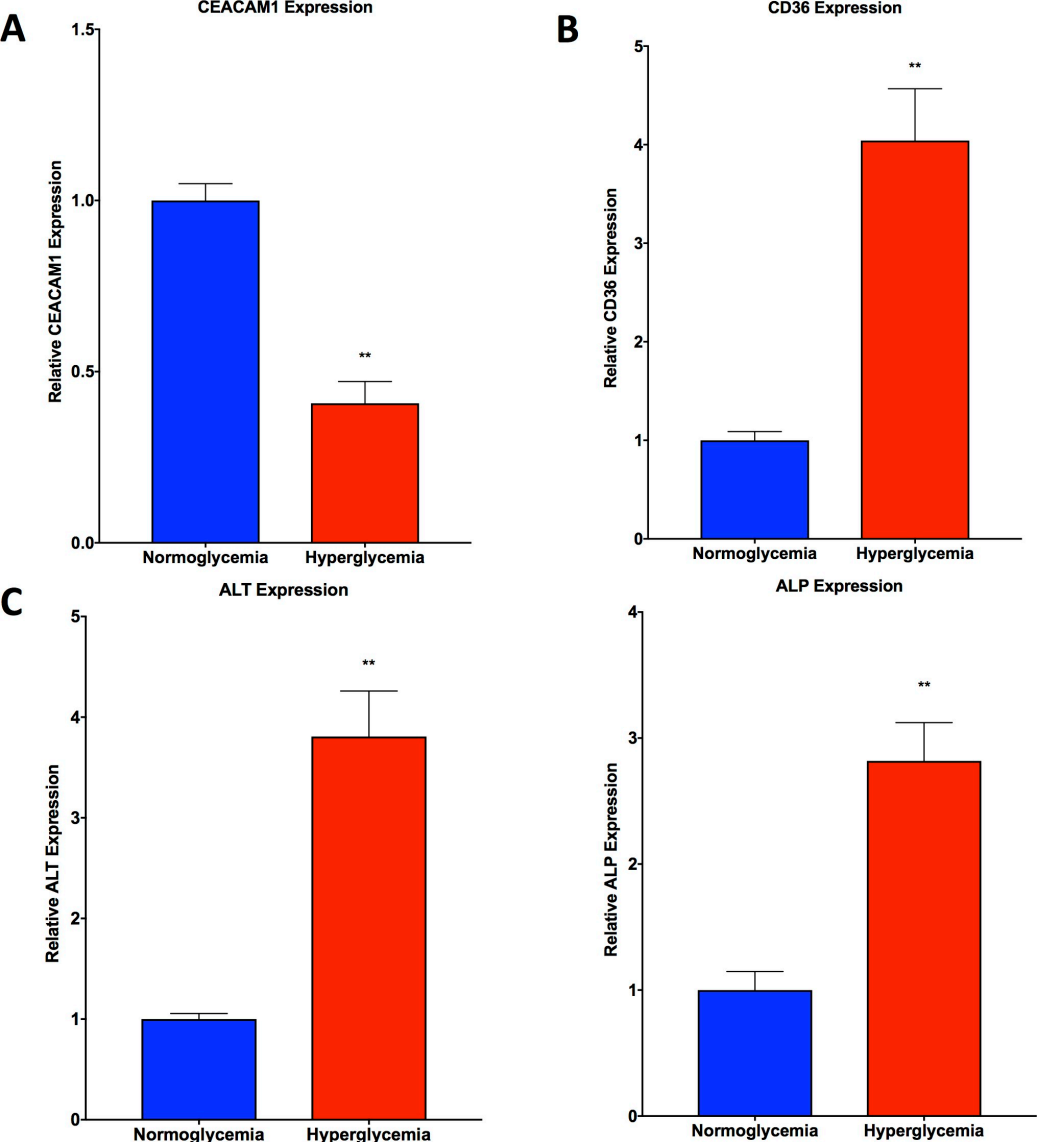
qPCR analysis shows trends in hyperglycemic HepG2 cells consistent with NAFLD. **A.** CEACAM1 gene expression is depressed in hyperglycemic HepG2 cells as compared with normoglycemic cells. **B.** CD36 gene expression is elevated in hyperglycemic HepG2 cells as compared with normoglycemic cells. **C.** ALT and ALP gene expression is elevated in hyperglycemic HepG2 cells as compared with normoglycemic cells. Data presented indicate the mean ± SEM (n = 3 per group). **p<0.01 vs. normoglycemic group.

qPCR analysis of CEACAM1 revealed a significant suppression of gene expression in hyperglycemic HepG2 cells compared with normoglycemic HepG2 cells (**Fig 3A**). CD36 gene expression was observed to be significantly upregulated in hyperglycemic HepG2 cells compared with normoglycemic HepG2 cells (**Fig 3B**). Similarly, ALT and ALP were both observed to be significantly upregulated in hyperglycemic HepG2 cells versus normoglycemic HepG2 cells (**Fig 3C**). Hepatotoxicity gene array data showed upregulation of SERPINE1, SCD, SREBF1, and THRSP, and downregulation of CYP1A2 and ABCC2 (**Table 1**).

**Table 1:** Hepatotoxicity gene array reveals regulation of key genes found in NAFLD.

Gene	HepG2 Normoglycemia vs Hyperglycemia	WT C57BL/6J vs Lepr^db^/J Mice
SERPINE1	11.78	12.74
SCD	6.72	148.14
SREBF1	5.97	8.44
THRSP	3.73	2.19
CYP1A2	-4.03	-37.08
ABCC2	-2.12	-33.73

Regulation of key genes that are characteristic to NAFLD are seen to be regulated in similar patterns *in vitro* and *in vivo*.

**Fig 4:**
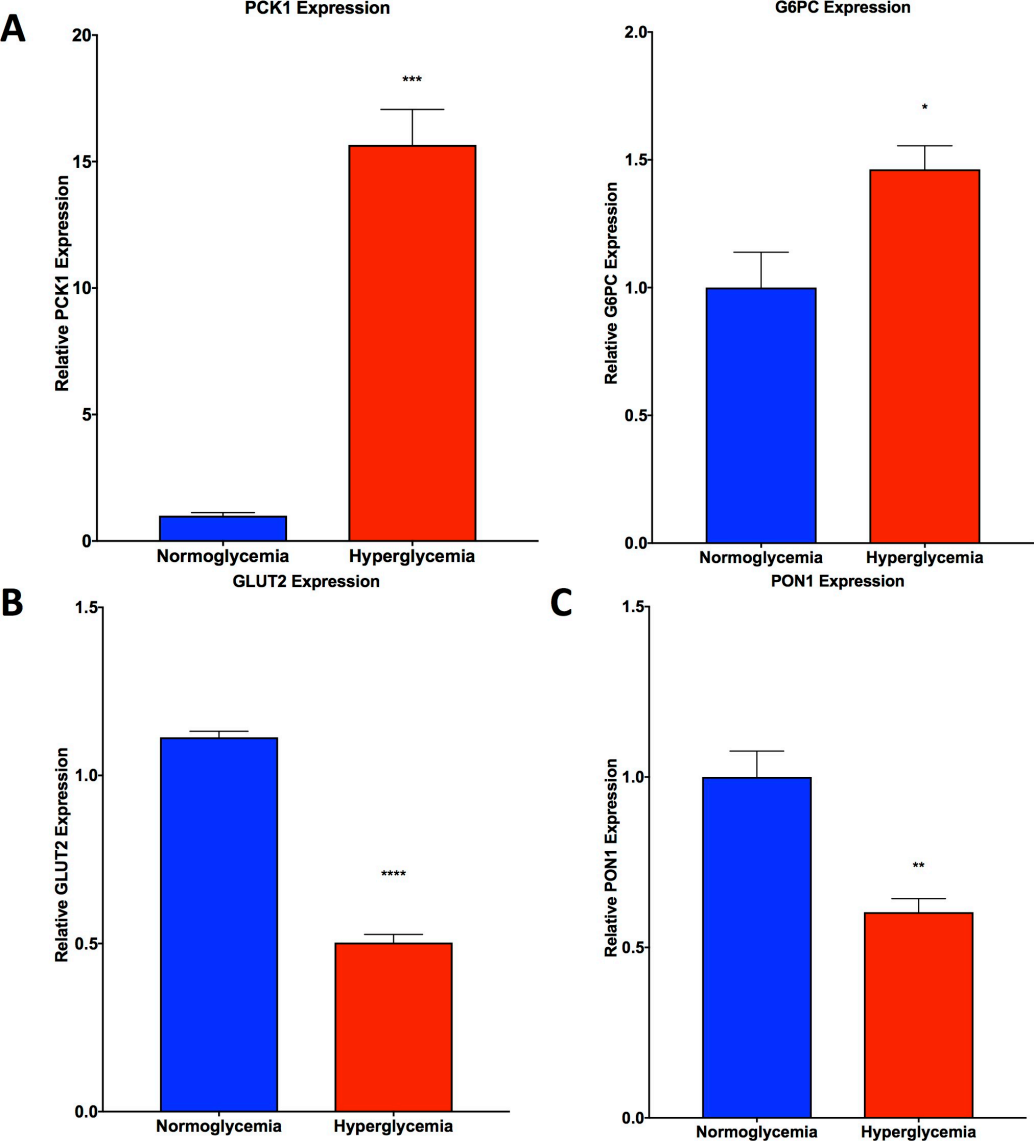
qPCR analysis shows trends in hyperglycemic HepG2 cells consistent with insulin resistance. **A.** PCK1 and G6PC gene expression is elevated in hyperglycemic HepG2 cells compared with normoglycemic HepG2 cells. **B.** GLUT2 and **C.** PON1 gene expression is depressed in hyperglycemic HepG2 cells compared with normoglycemic HepG2 cells. Data presented indicate the mean ± SEM (n = 3 per group). *p<0.05, ***p<0.001, ****p<0.0001 vs. normoglycemic group.

qPCR analysis of Phosphoenolpyruvate Carboxykinase 1 (PCK1) and Glucose-6-Phosphatase (G6PC) revealed elevated gene expression levels of both PCK1 and G6PC in hyperglycemic HepG2 cells as compared with normoglycemic HepG2 cells (**Fig 4A**). In addition, the gene expression of glucose transporter 2 (GLUT2, **Fig 4B**) and paraoxonase 1 (PON1, **Fig 4C**) was found to be diminished in hyperglycemic HepG2 cells as compared with normoglycemic cells.

In order to account for differences in osmolarity, an osmotic control was established by culturing HepG2 cells in normoglycemic media with osmolarity adjusted to that of hyperglycemic media using sodium chloride (NaCl). qPCR for select key genes was conducted to compare normoglycemic HepG2 cells with HepG2 cells cultured in normoglycemic media with adjusted osmolarity. qPCR analysis of ALT, ALP, CEACAM1, PCK1, G6PC, and GLUT2 revealed no significant differences in gene expression between normoglycemic HepG2 cells with HepG2 cells cultured in normoglycemic media with adjusted osmolarity (**S2 Fig**).

### Mitochondrial function and cell viability are unchanged in hyperglycemic HepG2 cells as compared with normoglycemic HepG2 cells

**Fig 5:**
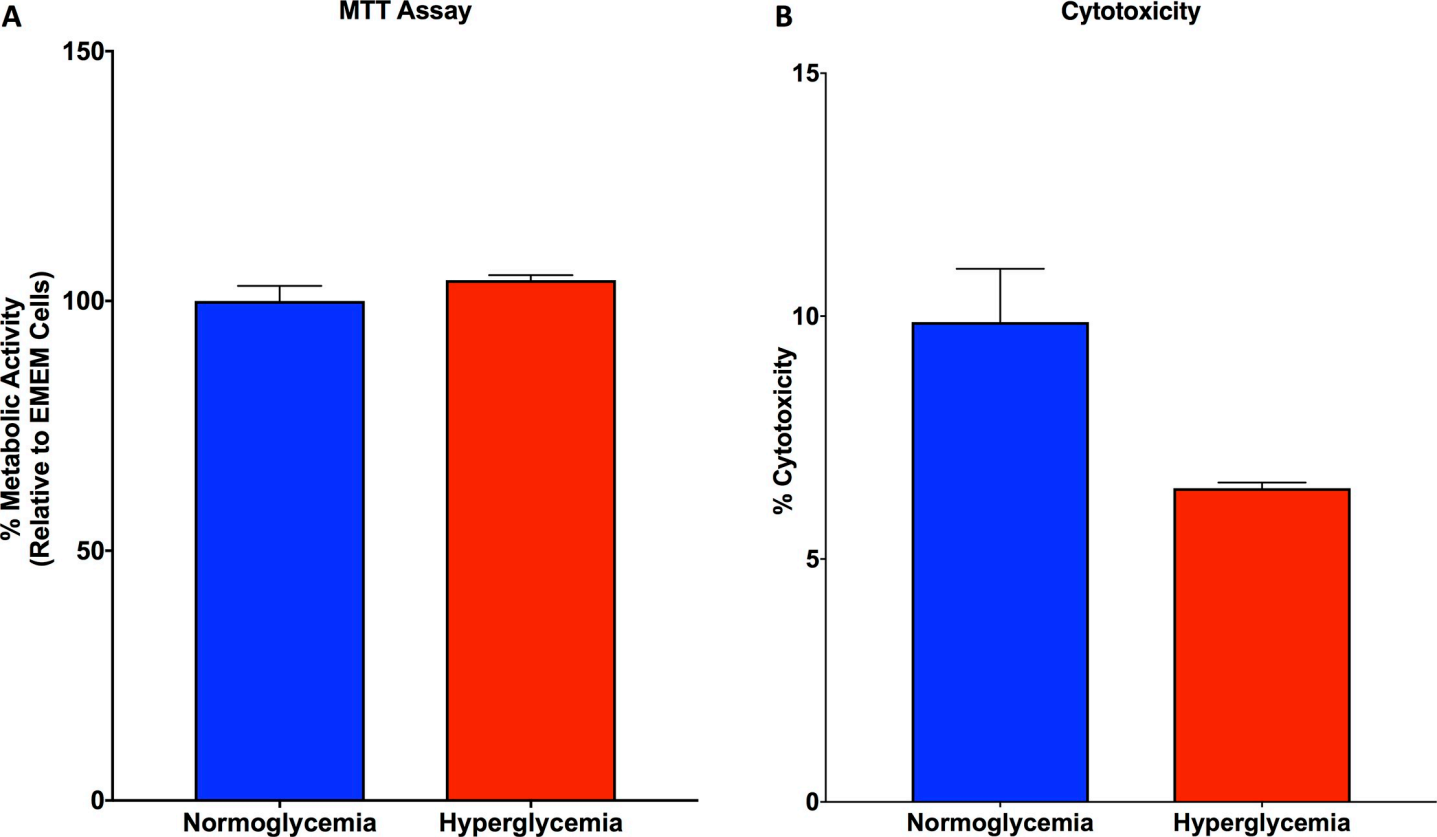
MTT and LDH assay for HepG2 cells reveal no significant differences between cell conditions. **A.** MTT assay demonstrated no differences in mitochondrial function between hyperglycemic and normoglycemic HepG2 cells. **B.** LDH assay demonstrated no differences in cytotoxicity between hyperglycemic and normoglycemic HepG2 cells.

MTT assay for mitochondrial function demonstrated that there was no significant change in function between hyperglycemic and normoglycemic HepG2 cells (**Fig 5A**). Similarly, LDH assay for cytotoxicity revealed no changes in cell death between hyperglycemic and normoglycemic HepG2 cells (**Fig 5B**).

### Lipid content is elevated in NAFLD mice compared with healthy WT mice

**Fig 6:**
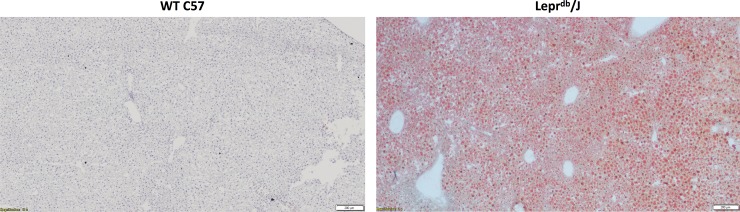
Oil Red O staining is elevated in livers of Lepr^db^/J mice. Livers from Lepr^db^/J mice revealed increased Oil Red O staining as compared with livers from WT C57 mice.

Oil Red O lipid staining of liver sections reveal visibly elevated accumulation of lipid within NAFLD livers of Lepr^db^/J mice on a C57BL/6J background as compared with healthy, WT C57BL/6J mice (**Fig 6**).

### Gene expression of key biomarkers observed in the mouse model reflect regulation consistent with that of NAFLD

**Fig 7:**
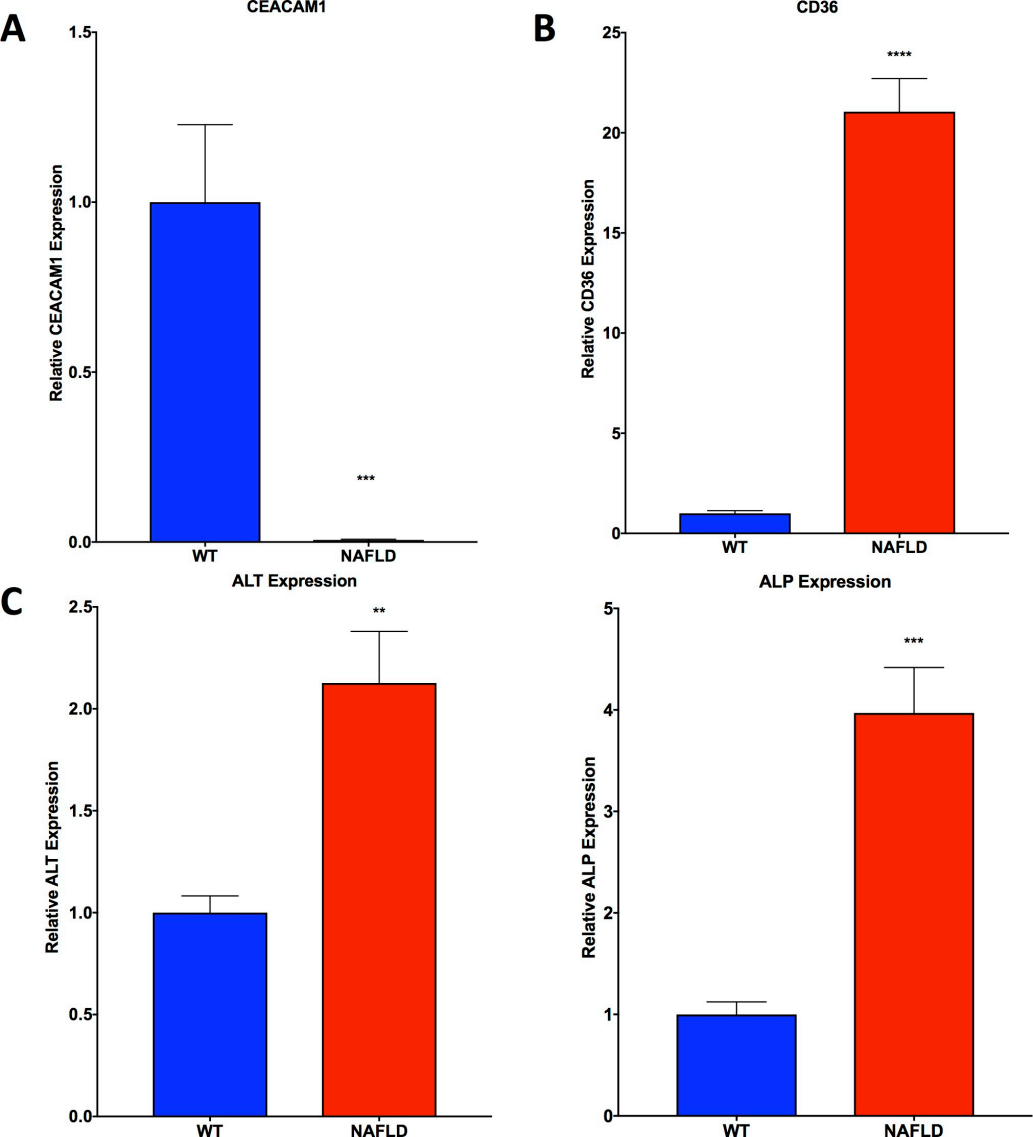
qPCR analysis shows trends in Lepr^db^/J mice consistent with NAFLD. **A.** CEACAM1 gene expression is depressed in Lepr^db^/J mouse livers as compared with WT C57 mouse livers **B.** CD36 gene expression is elevated in Lepr^db^/J mouse livers as compared with WT C57 mouse livers **C.** ALT and ALP gene expression is elevated in Lepr^db^/J mouse livers as compared with WT C57 mouse livers. Data presented indicate the mean ± SEM (n = 5–6 per group). **p<0.01, ***p<0.001, and ****p<0.0001 vs. WT C57 group.

Gene expression of CEACAM1 was found to be significantly downregulated in liver tissue from Lepr^db^/J mice on a C57BL/6J background as compared with healthy, WT C57BL/6J mice (**Fig 7A**). CD36 was found to be significantly upregulated in the NAFLD mice compared with healthy, WT mice (**Fig 7B**). Gene expression of ALT and ALP were found to be significantly upregulated in NAFLD mice compared with healthy, WT mice (**Fig 7C**). Hepatotoxicity gene array analysis showed regulation in line with what was observed *in* vitro, with upregulation of SERPINE1, SCD, SREBF1, and THRSP, and downregulation of CYP1A2 and ABCC2 (**Table 1**).

## Discussion

Non-alcoholic fatty liver disease (NAFLD) is a globally prevalent health concern that is an important link amongst a multitude of comorbidities, having been referred to as a “multisystem disease” [7]. NAFLD not only puts patients at risk of hepatic progression towards non-alcoholic steatohepatitis, cirrhosis, and hepatocellular carcinoma, but also serves as a mediator amongst diseases such as obesity, metabolic syndrome, diabetes, cardiovascular disease, and chronic kidney disease. Because there is an urgent need to address key knowledge gaps regarding molecular mechanisms and therapeutic approaches surrounding NAFLD, this study aimed to provide a novel *in vitro* model which recapitulated several key features of NAFLD and could be used to advance research in this area.

This in vitro model exploits one of the most prominent comorbidities associated with NAFLD: hyperglycemia. A recent meta-analysis of 17 separate studies involving 10,897 type 2 diabetes patients found a 54% prevalence of NAFLD within these patients [8]. The model we have developed simulates normoglycemic conditions with 1g/L glucose in cell culture media and simulates hyperglycemic conditions with 4.5g/L glucose in cell culture media.

NAFLD is histologically characterized by lipid deposition, specifically triglycerides, within the cytoplasm of hepatocytes [9]. Upon noticing progressive phenotypic changes in hyperglycemic HepG2 cells over several passages versus normoglycemic HepG2 cells by brightfield visualization and H&E staining, cells were further evaluated using transmission electron microscopy. This ultrastructural imaging revealed the pronounced increase in microvesicular lipid deposition and accumulation in hyperglycemic HepG2 cells versus normoglycemic cells.

Given that NAFLD is characterized by triglyceride deposition, Nile red was used in order to visualize and quantitatively measure triglyceride content, as Nile red is specific for neutral lipids such as triglyceride [10]. Not only do hyperglycemic HepG2 cells express more Nile red fluorescence intensity visually, but quantitative measurement of Nile red fluorescence normalized to protein concentration also confirms the enhanced triglyceride content within hyperglycemic HepG2 cells compared with normoglycemic HepG2 cells.

Alanine aminotransferase (ALT) and alkaline phosphatase (ALP) are common biomarkers of liver injury that are currently used clinically. These sensitive biomarkers are used to diagnose and monitor liver damage and disease due to a wide range of insults, including NAFLD [11]. In fact, NAFLD and alcoholic liver disease are the most common causes for the elevation of transaminases [12]. Through our qPCR analysis, we found an elevation in both ALT and ALP gene expression in hyperglycemic HepG2 cells as compared with normoglycemic cells, consistent with what is seen in NAFLD clinically.

Another key gene of interest in NAFLD is CD36. CD36 is also known as “fatty acid translocase” and has been associated with NAFLD. In fact, a hepatocyte-specific CD36 knockout model demonstrated an attenuation of hepatic steatosis and improved insulin sensitivity [13]. In accordance with this, CD36 was observed to be upregulated in hyperglycemic HepG2 cells as compared to normoglycemic HepG2 cells, serving as a potential mediator of fatty acid deposition and accumulation within steatotic hepatocytes. Similarly, paraoxonase-1 (PON1) is a liver derived antioxidant enzyme which is has been shown to be diminished in NAFLD [14–18] and was significantly decreased in hyperglycemic vs. normoglycemic HepG2 cells.

NAFLD is also characterized by the expressional regulation of other more specific key genes including CEACAM1. A decrease in CEACAM1 has been found to be a marker of insulin resistance, with its overexpression having been shown to reverse insulin resistance [19, 20]. CEACAM1 is involved in promoting insulin clearance and its loss in hepatic expression has been shown to link insulin resistance to obesity and NAFLD [21]. Our real-time PCR analysis of hyperglycemic HepG2 cells reveal suppression of CEACAM1 as compared to normoglycemic HepG2 cells, which suggests insulin resistance as a result of hyperglycemia and serves as a link to obesity and NAFLD.

Insulin resistance is a characteristic that manifests in conjunction with NAFLD. The expression of several key genes have become well accepted markers of insulin resistance. Phosphoenolpyruvate carboxykinase (PCK1), is a key enzyme in gluconeogenesis that catalyzes the first committed step in the overall process [22]. Glucose-6-phosphatase (G6PC) plays an equally important role in gluconeogenesis, as it catalyzes the final step in the overall process [23]. As a result of insulin resistance, gluconeogenesis is activated, which stimulates the increase in PCK1 and G6PC gene expression. A complete profiling of Lepr^db^/J mouse livers established that PCK1 and G6PC gene expression is elevated in the setting of type 2 diabetes as a result of insulin resistance [24]. Similarly, PCK1 and G6PC are also elevated in other models of diabetes in rodents, such as the streptozotocin-diabetes model in rats [23]. Other studies have found that PCK1 gene silencing can improve glycemic control, insulin sensitivity, and aberrant dyslipidemia in Lepr^db^/J mice. Interestingly, we have found that our model highlights this elevation in PCK1 and G6PC gene expression within hyperglycemic HepG2 cells as compared with normoglycemic HepG2 cells.

Another indicator of insulin resistance and NAFLD is the expression of glucose transporter 2 (GLUT2). It has been shown that in the high fat diet fed rat model of NAFLD induction, GLUT2 gene expression is found to be depressed [25]. Subsequent treatment with PPARδ agonists have been shown to reverse NAFLD pathogenesis by restoring glucose and fatty acid metabolism and restoration of GLUT2 gene expression. Our study shows that GLUT2 gene expression is depressed in hyperglycemic HepG2 cells as compared with normoglycemic HepG2 cells.

Although these data support the phenotypic and genetic characteristics of NAFLD in our *in vitro* model, we wanted to also compare and contrast these findings in an *in vivo* model of NAFLD. Lepr^db^/J mice have been used previously and are a well-established, genetic model of non-alcoholic fatty liver disease [26]. These mice were compared with healthy, wild type C57BL/6J mice. From Oil Red O lipid staining of hepatic tissue, microscopic visualization clearly shows a significant accumulation of lipid in the Lepr^db^/J mice as compared with the healthy, C57BL/6J mice, confirming the NAFLD phenotype of steatosis. Consistent with NAFLD, ALT, ALP, and CD36 are observed to be significantly upregulated in the Lepr^db^/J mouse livers as compared with the healthy, WT livers, which is also seen in the HepG2 model. Also consistent with NAFLD, CEACAM1 was found be significantly downregulated in the Lepr^db^/J mice. Interestingly, hepatotoxicity gene array analysis shows upregulation and downregulation of similar genes in both the mouse and cell models of NAFLD. These include the upregulation of SERPINE1, SCD, SREBF1, and THRSP, and downregulation of CYP1A2, and ABCC2. The upregulation and downregulation of these specific genes have been previously linked to NAFLD [27–32].

This current model of NAFLD induction in HepG2 cells exploits a physiologically relevant induction method, given that hyperglycemia commonly and progressively leads to a NAFLD comorbidity in humans. While this is a convenient method that models a relevant phenomenon, it has been previously observed that the use of saturated fatty acids, such as palmitic acid, can lead to a direct induction of lipid accumulation within HepG2 cells [33]. This direct induction method leads to a similar phenotypic and genetic profile as that observed with our model. Palmitic acid exposure leads to lipid accumulation within HepG2 cells as observed with Oil Red O staining [33]. Accompanying the observed steatosis, palmitic acid-induced lipid accumulation also leads to increased expression of CD36, SREBF1, and SCD1 [33, 34]. Other studies have found that primary hepatocytes exposed to fatty acids, such as palmitic acid, leads to a decrease in CEACAM1 expression as well as a decrease in GLUT2 [19, 35, 36]. The increase in steatosis, increased expression of CD36, SREBF1, and SCD1, and the decreased expression of CEACAM1 and GLUT2 are all key features also seen in our hyperglycemic HepG2 model as well as the Lepr^db^/J mouse model.

Together, these findings reveal that a hyperglycemic cell culturing method can be used to induce a novel *in vitro* model of steatosis and insulin resistance which is common in NAFLD. This model provides a rapid and simple protocol for inducing key genetic and phenotypic features of NAFLD, which is supported by key histopathological findings, the quantitatively determined deposition and accumulation of triglycerides, and the expressional regulation of key genes linked with NAFLD. These characteristics were were similar to a well-established *in vivo* model of NAFLD. We believe that characterization of this *in vitro* model will help to propel future studies in identifying key molecular mechanisms of NAFLD pathogenesis and in elucidating potential new therapies to attenuate the globally pervasive consequences of NAFLD.

### Limitations

HepG2 cells are a widely used human liver epithelial cell line and have been extensively used in studying different hepatic diseases and insults. However, although they are commonly utilized in *in vitro* studies, it is important to note that HepG2 cells differ in several key aspects as compared with primary hepatocytes. One of these differences is seen with the cytochrome P450 system. It has been previously reported that HepG2 cells express all of the major CYP enzymes, such as CYP1A1, 1A2, 2A6, 2B6, 2C8, 2C9, 2C19 [37], 2D6, 2E1, and 3A4, however, their mRNA expression levels were found to be much lower than those found in primary human hepatocytes. Therefore, while HepG2 cells can provide a beneficial and efficient *in vitro* model for a wide range of studies, results should be confirmed in *in vivo* models in order to account for differences in cell biology.

## Supporting information

S1 Table Normoglycemic versus hyperglycemic media ingredient comparison. (DOCX)

S1 Fig Brightfield imaging of HepG2 cells. HepG2 cells were passaged several times in either normoglycemic media, normoglycemic media with adjusted osmolarity, or hyperglycemic media. In the number of passages needed to induce fatty change in the hyperglycemic HepG2 cells, there were no phenotypic changes in the HepG2 cells with adjusted osmolarity, however, their phenotype remains similar to that of normoglycemic HepG2 cells.
(TIFF)

S2 Fig qPCR analysis between normoglycemic HepG2 cells and normoglycemic HepG2 cells with adjusted osmolarity. Gene expression of ALT, ALP, CEACAM1, PCK1, G6PC, and GLUT2 show no significant differences between normoglycemic HepG2 cells and normoglycemic HepG2 cells with adjusted osmolarity.
(TIFF)
