# Supplementary material for: Hyperglycemia induces key genetic and phenotypic changes in human liver epithelial HepG2 cells which parallel the Leprdb/J mouse model of non-alcoholic fatty liver disease (NAFLD)
Source: PLoS One. 2019 Dec 5;14(12):e0225604. doi: 10.1371/journal.pone.0225604 (PMC6894821; doi:10.1371/journal.pone.0225604)
Supplement: S1 Table — (DOCX) [file pone.0225604.s001.docx]

| **Component** | **EMEM (g/L)** | **DMEM (g/L)** |
| --- | --- | --- |
| Glucose | 1 | 4.5 |
| Sodium Pyruvate | 0.11 | 0.11 |
| Phenol Red | 0.01 | 0.015 |
| Calcium Chloride 2H2O | 0.2649 | 0.2 |
| Magnesium Sulfate | 0.0977 | 0.0977 |
| Potassium Chloride | 0.4 | 0.4 |
| Sodium Bicarbonate | 1.5 | 3.7 |
| Sodium Chloride | 6.8 | 6.4 |
| Sodium Phosphate Monobasic H2O | 0.14 | 0.125 |
| L-Alanine | 0.0089 | NA |
| L-Arginine HCl | 0.1264 | 0.084 |
| L-Asparagine H2O | 0.015 | NA |
| L-Aspartic Acid | 0.0133 | NA |
| L-Cystine 2HCl | 0.0312 | 0.0626 |
| L-Glutamic Acid | 0.0147 | NA |
| L-Glutamine | 0.292 | 0.584 |
| Glycine | 0.0075 | 0.03 |
| L-Histidine HCl H2O | 0.0419 | 0.042 |
| L-Isoleucine | 0.0525 | 0.1048 |
| L-Leucine | 0.0525 | 0.1048 |
| L-Lysine HCl | 0.0725 | 0.1462 |
| L-Methionine | 0.015 | 0.03 |
| L-Phenylalanine | 0.0325 | 0.066 |
| L-Proline | 0.0115 | NA |
| L-Serine | 0.0105 | 0.042 |
| L-Threonine | 0.0476 | 0.0952 |
| L-Tryptophan | 0.01 | 0.016 |
| L-Tyrosine 2Na 2H2O | 0.0519 | 0.1038 |
| L-Valine | 0.0468 | 0.094 |
