# Supplementary figures and images for: Hyperglycemia induces key genetic and phenotypic changes in human liver epithelial HepG2 cells which parallel the Leprdb/J mouse model of non-alcoholic fatty liver disease (NAFLD)
Source: PLoS One. 2019 Dec 5;14(12):e0225604. doi: 10.1371/journal.pone.0225604 (PMC6894821; doi:10.1371/journal.pone.0225604)

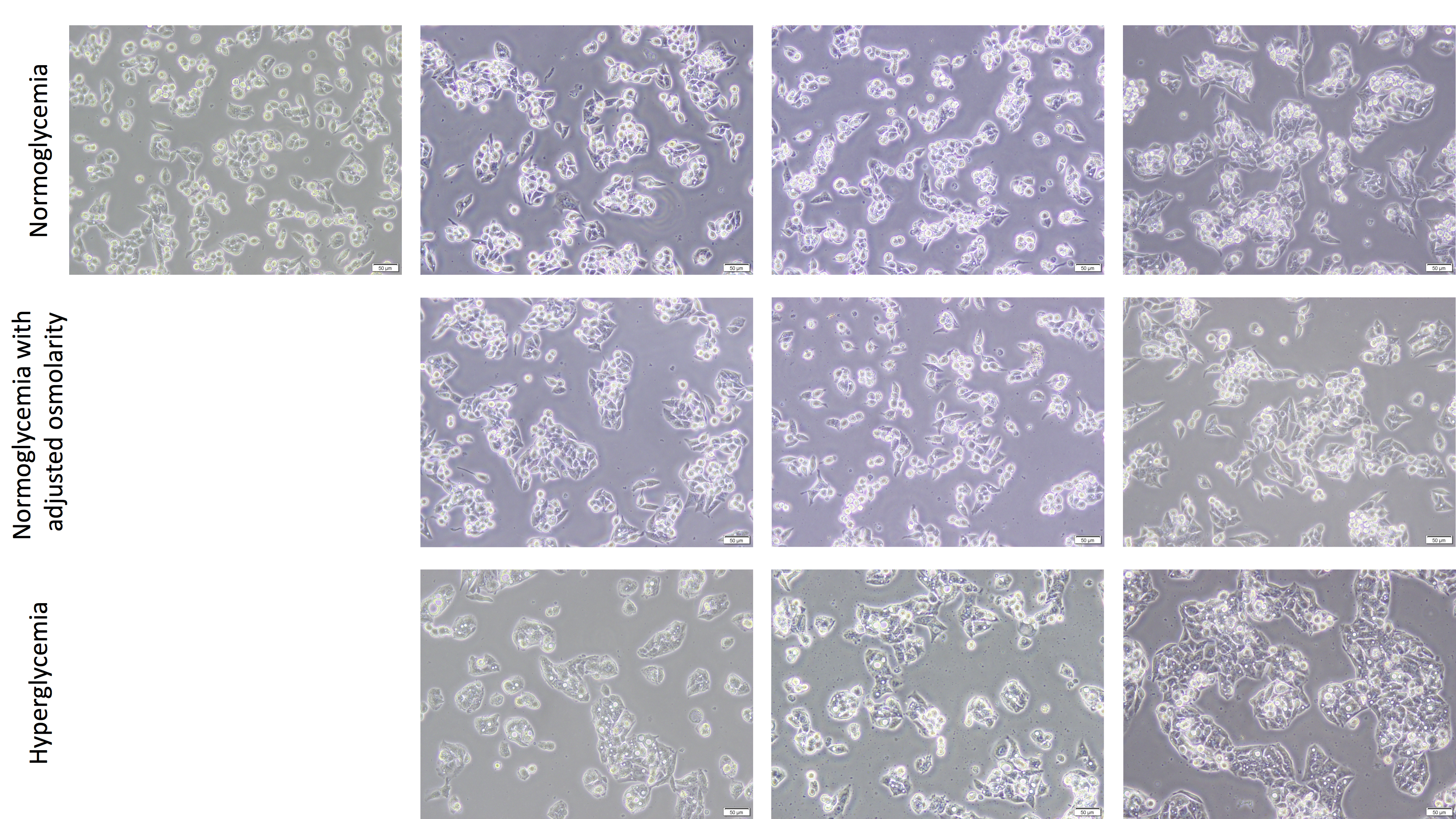

Supplement: S1 Fig — HepG2 cells were passaged several times in either normoglycemic media, normoglycemic media with adjusted osmolarity, or hyperglycemic media. In the number of passages needed to induce fatty change in the hyperglycemic HepG2 cells, there were no phenotypic changes in the HepG2 cells with adjusted osmolarity, however, their phenotype remains similar to that of normoglycemic HepG2 cells. (TIFF) [file pone.0225604.s002.tiff]

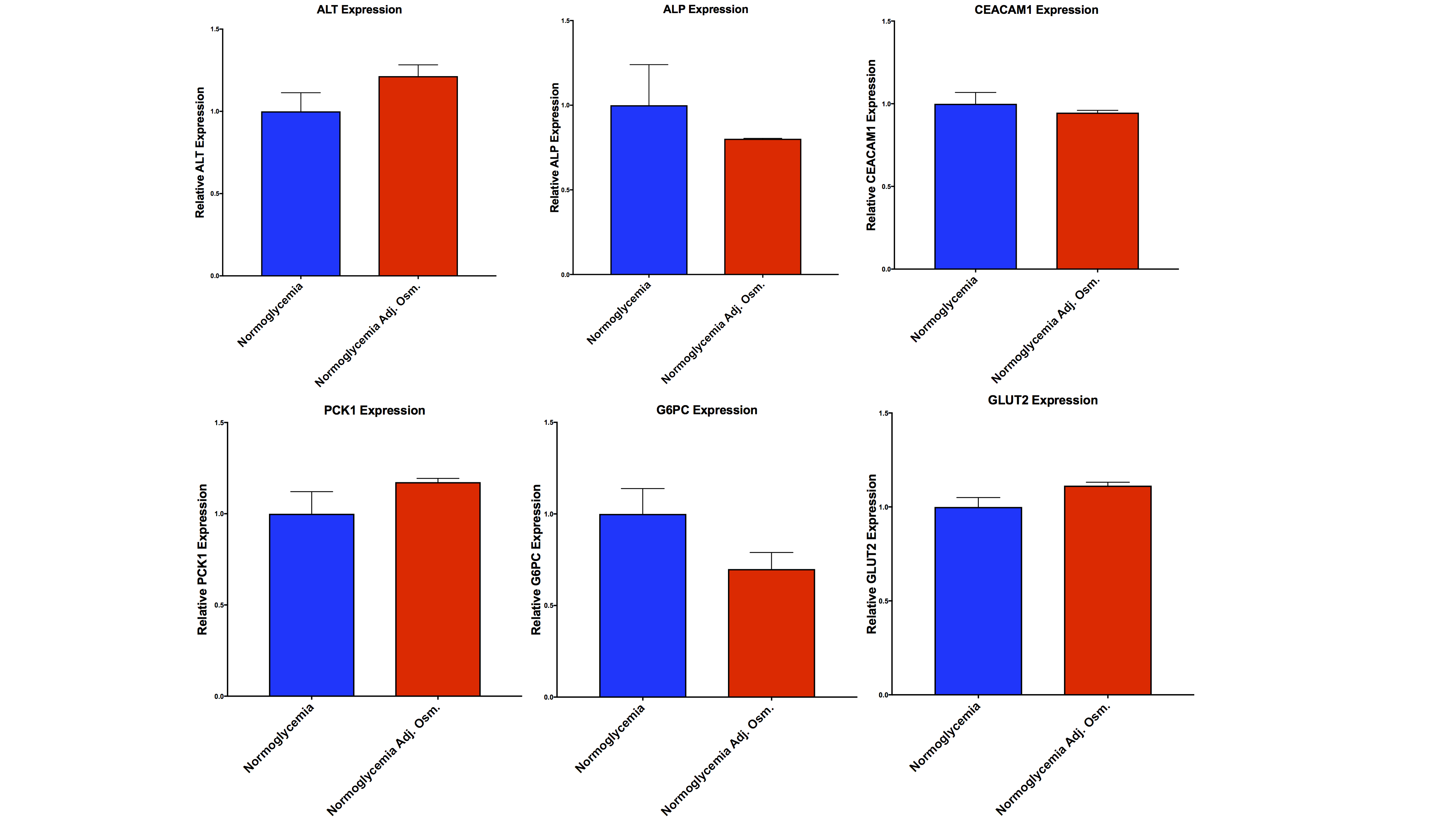

Supplement: S2 Fig — Gene expression of ALT, ALP, CEACAM1, PCK1, G6PC, and GLUT2 show no significant differences between normoglycemic HepG2 cells and normoglycemic HepG2 cells with adjusted osmolarity. (TIFF) [file pone.0225604.s003.tiff]
